# Low vaccination coverage of Greek Roma children amid economic crisis: national survey using stratified cluster sampling

**DOI:** 10.1093/eurpub/ckw179

**Published:** 2016-09-30

**Authors:** Dimitris Papamichail, Ioanna Petraki, Chrisoula Arkoudis, Agis Terzidis, Emmanouil Smyrnakis, Alexis Benos, Takis Panagiotopoulos

**Affiliations:** 1 Department of Child Health, National School of Public Health, Athens, Greece; 2 Laboratory of Primary Health Care, General Practice and Health Services Research, Department of Medicine, Aristotle University of Thessaloniki; 3 Programme “Education of Roma children”, Centre for Intercultural Studies, Faculty of Philosophy, Pedagogy and Psychology, University of Athens; 4 Programme of Postgraduate Education “International Medicine - Health Crisis Management”, Medical School, University of Athens

## Abstract

**Background:** Research on Roma health is fragmentary as major methodological obstacles often exist. Reliable estimates on vaccination coverage of Roma children at a national level and identification of risk factors for low coverage could play an instrumental role in developing evidence-based policies to promote vaccination in this marginalized population group. **Methods**: We carried out a national vaccination coverage survey of Roma children. Thirty Roma settlements, stratified by geographical region and settlement type, were included; 7–10 children aged 24–77 months were selected from each settlement using systematic sampling. Information on children’s vaccination coverage was collected from multiple sources. In the analysis we applied weights for each stratum, identified through a consensus process. **Results:** A total of 251 Roma children participated in the study. A vaccination document was presented for the large majority (86%). We found very low vaccination coverage for all vaccines. In 35–39% of children ‘minimum vaccination’ (DTP3 and IPV2 and MMR1) was administered, while 34–38% had received HepB3 and 31–35% Hib3; no child was vaccinated against tuberculosis in the first year of life. Better living conditions and primary care services close to Roma settlements were associated with higher vaccination indices. **Conclusions:** Our study showed inadequate vaccination coverage of Roma children in Greece, much lower than that of the non-minority child population. This serious public health challenge should be systematically addressed, or, amid continuing economic recession, the gap may widen. Valid national estimates on important characteristics of the Roma population can contribute to planning inclusion policies.

## Introduction

The 2008–09 economic crisis affected a large number of countries globally. Greece is experiencing a particularly deep and long-lasting economic recession.^1^ As in other most affected countries, the situation of families has deteriorated, mostly from job loss, underemployment and cuts to public services, while the poorest and most vulnerable children have suffered disproportionately and inequalities have increased.^2^ Roma children are a particularly vulnerable social group.

Roma people are dispersed throughout Europe, representing its largest ethnic minority. An estimated 11 million Roma live in Europe.^3^ Estimates of the Roma population in Greece range from 120 000–300 000.^4^

### Roma health

When compared with majority populations, Roma experience more poverty, unemployment, substandard housing, illiteracy as well as social exclusion.^5^^,^^6^ It has been shown that Roma populations have shorter life expectancy, poorer health, and more barriers to health care.^7^^,^^8^ In particular, vaccination coverage studies of Roma children, have consistently shown low immunization rates.^9–12^

Systematic reviews on Roma health conducted in the last 15 years have pointed out that published research is sparse and fragmentary, in many instances lacking methodological rigour.^7^^,^^13^^,^^14^ Similarly, studies on Roma health in Greece are scarce and have essential methodological limitations.^15^^,^^16^ Lack of valid records of Roma settlements in Greece and absence of accurate estimates of their population size pose important obstacles to conducting studies on Roma health.

### Vaccination in Greece

Roma children are immunized according to the National Vaccination Programme (NVP) of Greece (Supplementary Table S1 and Box 1).^17^ Vaccines are provided free of charge to residents of the country, and are typically recorded on the Child Health Booklet which has been shown to be accurately completed and maintained by the parents of the vast majority of children (around 98%) in the general population.^18^ Results of the 2012 national vaccination coverage survey among 6-year-old children of the general population, showed high immunization rates for most of the vaccines included in the NVP and identified several socioeconomic factors and structural barriers as determinants of poor vaccination.^19^

**Box 1:** Abbreviations of vaccine names

BCG:	Bacillus Calmette-Guérin vaccine (against tuberculosis)
DTP:	Diphtheria-tetanus-pertussis vaccine
HepA:	Hepatitis A vaccine
HepB:	Hepatitis B vaccine
Hib:	Haemophilus influenzae type b vaccine
IPV:	Inactivated poliomyelitis vaccine
MCVC:	Meningococcal conjugate vaccine, serogroup C
MMR:	Measles-mumps-rubella vaccine
PCV:	Pneumococcal conjugate vaccine
Var:	Varicella vaccine
*Note:*	Number after vaccine name denotes doses (e.g. MMR2: two doses of MMR vaccine)

Published research on vaccination coverage of Roma children in Greece is limited, and current knowledge is based on anecdotal information, small community-level studies, or social studies which focus on other issues and only marginally refer to vaccination.^15^^,^^20^^,^^21^ In Greece, most Roma children are vaccinated in Health Centers, which provide primary care mainly in rural areas, hospital outpatient clinics, and Medical-Social Centers, which started operating in 2006 close to settlements in order to support Roma integration and experienced serious underfunding in the years of the economic crisis.

Reliable estimates of Roma vaccination coverage at a national level in Greece could play an instrumental role in developing evidence-based policies, advocating for the appropriate priority of measures to promote vaccination among this population of children, and averting disproportionate ill effects of the economic crisis on Roma vaccination.

### Aim of the study

The aim of this study was to obtain reliable quantitative estimates of vaccination coverage of the Greek Roma child population at a national level, and identify risk factors regarding Roma settlement characteristics associated with low immunization coverage.

## Methods

In 2012–13 we carried out a cross sectional study of the Greek Roma child population 24–77 months old, applying stratified cluster sampling. This was a modification of the 30 by 7 sampling design developed by WHO.^22^

### Sampling design

We divided the Greek Roma population in strata based on geographical region of residence and settlement type (Supplementary Table S2).^20^^,^^23^^,^^24^

We selected a total of 30 Roma settlements (clusters of children). In order to overcome the existing lack of valid records of Roma settlements in Greece and their population size (i) we specified the number of clusters (settlements) to be included in the sample from each region on the basis of population size and within-region geographical variation, (ii) within each region, we included an equal number of clusters from all settlement types and (iii) we used judgmental sampling of clusters in each stratum (region and settlement type) to ensure geographical dispersion of the sample and selection of typical settlements. The inclusion criterion for Roma settlements was having a population of 15 families or more; Roma families integrated in the urban tissue and non-Greek Roma families were excluded. Within selected settlements we conducted systematic sampling of 7–10 children aged 24–77 completed months who were gathered for a major event which was well organized in advance together with Roma community leaders and, among others, included comprehensive medical examination of children.

### Information collected

Information on vaccination status was collected from multiple sources: (i) From vaccination documents (Child Health Booklet or vaccination card), if available, which were inspected by trained health professionals of the study team; vaccines and doses carried out by key ages were recorded on a special form; ii) From parents/guardians by means of interview using a standard questionnaire; if a vaccination document (see (i) above) was available, the parent was asked if additional vaccines or doses which were not recorded on the document had been administered to the child; if a vaccination document was not available, the parent was asked his/her subjective view on the child’s vaccination (no vaccine at all, ‘a small number’ or ‘an adequate number’ of vaccines); (iii) From records kept by local health services, if available; (iv) Specifically for BCG, field investigators looked for the characteristic scar in the arm.

Information on perceived barriers to vaccination (inadequate information, financial reasons, access difficulties, family issues) was collected by interviewing parents/guardians using a structured questionnaire. Information on the main characteristics of settlements (number of families, availability of electricity, water supply and sewage system, proximity to health services and educational facilities) was gathered by interviewing two to four key persons from each settlement (Roma community leaders and non-Roma professionals from local services) using a structured questionnaire and forming consensus opinion. All questionnaires used were previously piloted in two Roma settlements not included in the sample.

### Statistical analysis

We carried out data entry using Epidata (Epidata association, Denmark, version 3.1). Consistency and range checks were performed for data validation. The analysis was carried out using STATA (Stata Corporation, TX, USA, version 11).

In the analysis we applied sampling weights for each stratum (Supplementary Table S2). As weights we used estimates of the relative size of each stratum (proportion of the Greek Roma population living in each one), based on information from previous studies,^4^^,^^16^^,^^23^^,^^24^ and expert consultation. Five experts in the field of Roma issues in Greece were involved who were given all available information and were asked to make independent estimates of the proportion of the Roma population in each first level of Nomenclature of Units for Territorial Statistics (NUTS-1) region and in each settlement type within the regions; subsequently a single estimate was reached through a consensus process. In the analysis we also took into account that cluster sampling had been implemented.

In order to assess the overall immunization status of the study children we developed compound vaccination indices: ‘at least one vaccine dose’: at least one dose of any vaccine; ‘minimum vaccination’: all of DTP3, IPV2, MMR1; ‘basic vaccination’: all of DTP3, IPV2, MMR1, HepB2, Hib2; ‘extended vaccination’: all of DTP4, IPV3, MMR1, HepB3, Hib3, MCVC1, PCV1. We calculated high and low estimates of vaccination coverage (table 1). For high estimates we only took into account children who presented with a vaccination document; we considered they were immunized with the vaccines and doses recorded. For low estimates, in addition to the previous, we also took into account children without a vaccination document whose parent/guardians stated that they had had no vaccine at all (we considered them unvaccinated for all vaccines and doses studied). In the analysis for potential risk factors (tables 2 and 3) we used low immunization coverage estimates, which probably approximate more closely the actual coverage.

**Table 1:** Estimates of vaccination coverage: compound indices and coverage for selected doses of specific vaccines

Vaccine	Number of doses	Estimates of vaccination coverage Weighted percent (%)^a^ (95% CI)^b^	
		Low estimate^c^	High estimate^c^
		n = 240	n = 213
**Compound vaccination indices**			
At least one vaccine dose		80.8 (71.1–87.8)	90.3 (83.5–94.5)
Minimum vaccination		35.1 (25.4–46.2)	39.2 (29.0–50.5)
Basic vaccination		27.7 (18.7–38.9)	31.0 (21.4–42.5)
Extended vaccination		13.8 (7.1–25.0)	15.4 (8.1–27.4)
**Established vaccines**			
DTP	≥3	51.0 (37.8–64.1)	57.0 (43.6–69.5)
	≥4	26.8 (18.3–37.3)	29.9 (21.3–40.2)
	3 by age 12 months	19.9 (13.1–29.2)	22.3 (15.0–31.7)
IPV	≥2	61.0 (49.0–71.7)	68.2 (57.7–77.1)
	≥3	50.1 (37.9–62.4)	56.0 (43.7–67.6)
	2 by age 12 months	35.0 (23.2–49.0)	39.2 (27.0–52.8)
BCG	≥1	0.2 (0.0–1.5)	0.2 (0.0–1.6)
	1 by age 12 months	0	0
HepB	≥2	48.0 (37.4–58.8)	53.7 (43.3–63.8)
	≥3	33.8 (24.0–45.2)	37.8 (27.7–49.1)
	2 by age 12 months	26.1 (16.9–38.0)	29.2 (19.4–41.3)
Hib	≥3	30.9 (21.2–42.6)	34.5 (24.4–46.2)
	≥4	16.6 (9.8–26.7)	18.6 (11.3–28.9)
	3 by age 12 months	14.7 (9.5–22.1)	16.4 (10.9–23.9)
MMR	≥1	42.9 (33.7–52.6)	47.9 (38.4–57.6)
	≥2	6.8 (3.6–12.5)	7.6 (4.1–13.9)
	1 by age 24 months	18.8 (12.7–27.1)	21.1 (14.6–29.3)
**Vaccines introduced after 2005**			
MCVC	≥2	12.9 (6.3–24.4)	14.4 (7.3–26.5)
	≥3	6.3 (2.3–15.9)	7.0 (2.6–17.4)
	2 by age 12 months	7.6 (3.0–17.7)	8.5 (3.4–19.3)
PCV	≥3	16.3 (9.9–25.8)	18.3 (11.5–27.8)
	≥4	4.4 (2.3–8.1)	4.9 (2.7–8.8)
	3 by age 12 months	6.4 (3.7–10.9)	7.2 (4.3–11.8)
Var	≥1	24.7 (16.8–34.8)	27.6 (19.5–37.5)
	1 by age 24 months	8.9 (3.7–19.8)	10.0 (4.3–21.6)
HepA	≥1	22.6 (16.4–30.2)	25.2 (18.3–33.7)
	≥2	11.2 (7.3–17.2)	12.7 (8.5–18.7)
	2 by age 24 months	1.7 (0.5–5.7)	1.9 (0.6–6.2)

a: Percentages (%) are weighted for region (first level of Nomenclature of Units for Territorial Statistics, NUTS-1) and settlement type (according to the sampling design, these two variables define strata—see text); cluster design is also taken into account in the analysis.

b: 95% CI: 95% confidence interval. Although non-probability sampling was used in the first stage (selection of settlements), 95% CIs are presented in order to give a measure of imprecision of the estimates due to sampling.

c: High estimates were calculated taking into account only children who presented with a vaccination document and considering they were immunized for the vaccines and doses recorded. Low estimates were calculated taking into account, in addition to the previous category, children without a vaccination document whose parent/guardian stated that they had had no vaccine at all and considering they were unvaccinated for all vaccines and doses (see text).

**Table 2:** Vaccination indices by selected predictive factors (bivariate analysis)

Predictive factor	Vaccination document	At least one vaccine dose	Minimum vaccination	Basic vaccination	Extended vaccination	MMR1
	Weighted percent (%)^a^ (95% CI) ^b^	Weighted percent (%)^a^ (95% CI)^b^	Weighted percent (%)^a^ (95% CI)^b^	Weighted percent (%)^a^ (95% CI)^b^	Weighted percent (%)^a^ (95% CI)^b^	Weighted percent (%)^a^ (95% CI)^b^
**Gender**	*n* = 250	*n* = 239	*n* = 239	*n* = 239	*n* = 239	*n* = 239
Male	89 (81–94)	79 (70–86)	31 (20–44)	26 (17–37)	17 (10–29)	38 (27–51)
Female	83 (67–92)	83 (67–92)	39 (27–52)	30 (18–44)	11 (4–23)	48 (37–58)
*P* value	0.3143	0.5779	0.2558	0.5160	0.0587	0.1706
**Type of settlement**	*n* = 251	*n* = 240	*n* = 240	*n* = 240	*n* = 240	*n* = 240
Mainly houses (Type 1)	97 (91–99)	92 (82–97)	44 (26–63)	39 (22–58)	17 (5–44)	45 (28–64)
Houses and shacks/tents (Type 2)	77 (59–88)	74 (56–86)	28 (15–46)	22 (13–34)	7 (3–16)	35 (24–49)
Mainly shacks/tents (Type 3)	85 (68–94)	77 (61–88)	34 (20–51)	23 (10–45)	17 (6–38)	48 (33–63)
*P* value	0.0304	0.0742	0.3984	0.2513	0.4500	0.4666
**Region (NUTS-1)**	*n* = 251	*n* = 240	*n* = 240	*n* = 240	*n* = 240	*n* = 240
North Greece	93 (75–98)	87 (71–95)	37 (22–54)	31 (19–47)	14 (5–33)	44 (31–59)
Central Greece	78 (59–89)	76 (60–86)	36 (20–55)	25 (10–49)	15 (5–38)	44 (29–61)
Attica	84 (76–89)	73 (48–89)	29 (18–44)	22 (12–37)	10 (3–24)	37 (23–54)
Crete and Aegean islands	73 (41–91)	59 (32–81)	25 (12–46)	24 (12–42)	21 (12–35)	25 (12–46)
*P* value	0.1420	0.2914	0.7835	0.6330	0.8377	0.7886
**Size of settlement**	*n* = 251	*n* = 240	*n* = 240	*n* = 240	*n* = 240	*n* = 240
<100 families	88 (75–95)	79 (67–87)	32 (20–48)	21 (11–37)	10 (4–26)	41 (28–56)
≥100 families	84 (67–93)	83 (65–93)	38 (25–54)	35 (23–50)	18 (8–36)	45 (33–57)
*P* value	0.6467	0.6104	0.5612	0.1532	0.3936	0.7295
**Electricity available to ≥ 50% of houses in the settlement**	*n* = 251	*n* = 240	*n* = 240	*n* = 240	*n* = 240	*n* = 240
Yes	89 (78–95)	84 (75–91)	37 (26–49)	29 (19–42)	15 (8–28)	45 (34–55)
No	73 (52–87)	62 (39–81)	27 (14–45)	19 (9–35)	6 (2–21)	34 (18–55)
*P* value	0,0615	0,0312	0,3369	0.2660	0,2001	0,3313
**Usual service of vaccination**	*n* = 215	*n* = 207	*n* = 207	*n* = 207	*n* = 207	*n* = 207
Hospital	74 (57–86)	69 (54–81)	31 (18–48)	27 (15–45)	17 (7–37)	38 (25–53)
Primary Health Centre	93 (80–98)	86 (53–97)	56 (39–72)	40 (13–74)	27 (9–60)	59 (42–73)
Medical-Social Centre	95 (85–98)	89 (75–96)	37 (19–58)	26 (13–45)	2 (0–10)	52 (40–63)
*P* value	0,0027	0,1282	0,1665	0.6313	0,0751	0,1032
**Distance to usual vaccination service**	*n* = 251	*n* = 240	*n* = 240	*n* = 240	*n* = 240	*n* = 240
≤2Km	93 (87–97)	92 (84–96)	46 (34–59)	34 (20–51)	15 (5–36)	51 (42–61)
>2Km	80 (64–90)	71 (57–82)	25 (15–39)	22 (13–36)	13 (6–27)	36 (24–50)
*P* value	0,0159	0,0015	0,0226	0.2321	0,8590	0,0676
**Self-reported vaccination barrier**	*n* = 240	*n* = 229	*n* = 229	*n* = 229	*n* = 229	*n* = 229
No	86 (77–92)	88 (78–94)	45 (32–59)	32 (19–48)	16 (8–30)	54 (42–65)
Yes	83 (69–92)	72 (57–83)	23 (11–40)	19 (9–34)	6 (2–19)	31 (19–46)
*P* value	0.5628	0.0098	0.0343	0.1467	0.0666	0.0163

a: Percentages (%) are weighted for region (first level of Nomenclature of Units for Territorial Statistics, NUTS-1) and settlement type (according to the sampling design, these two variables define strata—see text); cluster design is also taken into account in the analysis. Low vaccination estimates are used in this analysis (see text).

b: For clarification on 95% CIs, see notes in table 1.

**Table 3:** Predictive factors for selected vaccination indices from logistic regression analysis (final models)

Predictive factor	Vaccination document	Extended vaccination	MMR1
	Adjusted RR^a^ (95%CI)^b^	Adjusted RR^a^ (95%CI)^b^	Adjusted RR^a^ (95%CI)^b^
	*n* = 251	*n* =251	*n* = 251
**Gender**	NA		NA
Male		Ref.	
Female		0.68 (0.42–1.09)	
**Type of settlement**		NA	NA
Mainly houses (Type 1)	Ref.		
Houses and shacks/tents (Type 2)	0.77 (0.64–0.94)		
Mainly shacks/tents (Type 3)	0.96 (0.91–1.01)		
**Electricity available to ≥ 50% of settlements’ houses**		NA	NA
Yes	Ref.		
No	0.76 (0.61–0.96)		
**Kindergarten close to the settlement**^c^	NA	NA	
Yes			Ref.
No			0.57 (0.47–0.70)
**Usual place of vaccination: Medical-Social Centre**	NA		NA
No		Ref.	
Yes		0.12 (0.02–0.59)	
**Self-reported barrier to vaccination**	NA	NA	
No			Ref.
Yes			0.57 (0.36–0.90)

a: Adjusted risk ratio.

b: For clarification on 95% CIs, see notes in table 1.

c: This variable remained in the final model, but only in one cluster (settlement) a kindergarten was not close to the settlement; therefore, it was decided not to further discuss this finding.

We carried out multiple logistic regression to identify factors independently associated with immunization status. As outcome variables we used (i) the compound vaccination indices mentioned earlier, (ii) availability of a vaccination document, (iii) vaccination with MMR1. Initial regression models were constructed including all variables for which the *P* value in the bivariate analysis was less than 0.1; gender, settlement type and NUTS-1 region were also included. We removed variables one at a time from the initial models on the basis of significance testing (*P* < 0.05) with the likelihood ratio test. The adjusted risk ratios derived from binomial regression using all the variables of the final logistic regression models are presented.

### Ethics

Ethical approval was obtained from the Bioethics Committee of the National School of Public Health, Greece (Decision no. 200/2012). Parents/guardians of children participating in the study provided verbal informed consent.

## Results

A total of 251 Roma children aged 24–77 months participated in the study. The median age was 4.2 years (interquartile range 3.0–5.2), and 50% were boys.

A vaccination document was presented for the large majority of children (213/251, weighted percentage 86.0%, 95% CI: 76.2–92.2%). Parents/guardians (mainly mothers) reported that the vaccination document accurately recorded the vaccines and doses administered to their child for all but eight children, for whom it was stated that they had had additional vaccines/doses. The parents/guardians of the 38 children lacking a vaccination document reported that 27 of them had received no vaccine at all, 10 had received a ‘small number’ of vaccines and one an ‘adequate number’. Vaccination records of Roma children were found only in four local health services and did not provide any additional information.

### Vaccination coverage

In about 80–90% of children ‘at least one vaccine dose’ had been administered; 35–40% had had ‘minimum vaccination’, around 30% ‘basic vaccination’ and around 15% ‘extended vaccination’. Table 1 shows the estimates of immunization coverage for specific vaccines and doses.

A little more than half of the children had been immunized with DTP3 and IPV3; one in five had received DTP3 and around one in three had had IPV2 by the age of 12 months.

About half of the children were immunized with two doses of HepB and around half of them had received them by the age of 12 months. A little less than half of the children were vaccinated with at least one dose of MMR and < 1 in 10 with two doses. Of note, only one child was found to be immunized with BCG, and in this case the vaccine was given after the first year of life. The characteristic BCG scar was detected only in this child (1/243), while for eight children this information was missing.

### Predictive factors

In bivariate analysis (table 2), characteristics indicating better housing (i.e. settlement mainly consisting of houses, electricity available to half or more of the houses in a settlement) were associated with presenting a vaccination document and initiating vaccination (immunized with at least one dose of any vaccine). Proximity (≤2 km) of the settlement to the service usually providing vaccination was also positively associated with these outcomes as well as with ‘minimum vaccination’. Parental reporting of barriers to vaccination tended to have a negative association with several vaccination coverage indices.

Children from settlements where vaccination is usually provided by a primary care service (Health Centre or Medical-Social Centre)—and not a hospital—were more likely to have a vaccination document and get at least one vaccine dose. Nevertheless, children from settlements where vaccination was usually provided by Medical-Social Centres were less likely to get ‘extended vaccination’ (table 2).

In multivariable analysis (table 3), staying in a settlement mainly consisting of houses and in one where electricity is available to half or more of the houses were independently associated with presenting a vaccination document. Being a girl and living in a settlement where vaccination is usually provided by a Medical-Social Centre were shown to have negative association with ‘extended vaccination’. Coverage with one dose of MMR was found to be less likely in children whose parents reported vaccination barriers. None of the predictive factors included in the multiple regression models we constructed was found to be associated with ‘at least one vaccination dose’, ‘minimum’ and ‘basic’ vaccination.

## Discussion

To our knowledge, this is the first published vaccination coverage survey of Roma children carried out at a national level. National estimates of vaccination coverage can provide the necessary evidence base for promoting comprehensive policies to meet vaccination needs of the Roma community in a country, but lack of records of Roma settlements, which is often the case,^25^^,^^26^ are obstacles for constructing sampling frames and deter such an endeavour. In order to overcome these obstacles, we implemented a study design involving cluster sampling with stratification and we used as weights in the analysis the relative size of strata, identified through a consensus process.

The large majority of children (86%) presented a vaccination document while very low vaccination coverage was found for all vaccines. Better living conditions and primary care services close to Roma settlements were positively associated with higher vaccination indices.

A limitation of this study is the non-probability sampling of clusters within strata, which may introduce selection bias. Nevertheless, this was an inevitable choice given the lack of reliable records of Roma settlements. The large number of strata employed probably tends to reduce this effect. Furthermore, the questionnaires used were piloted for simplicity and clarity, but no formal validation was carried out.

Our finding that more than eight out of ten children had a vaccination document available contradicts the widespread stereotypical perception that families of Roma children usually do not keep their vaccination booklet. Our finding is compatible with that of two national studies on Roma living conditions in Greece, conducted in 2000 and 2008, which found that for 72–78% of children Health Booklets were kept.^20^ A study of vaccination coverage in disadvantaged Roma settlements in Belgrade found that this was the case for 88% of the children.^9^

We found very low vaccination coverage for all vaccines in the Roma child population, e.g. coverage with DTP3 was 51–57%, IPV3 50–56%, HepB3 34–38%, MMR1 43–48%. Coverage of 6-year-old children in the non-minority population in Greece was 98 to >99% for all these vaccine doses, according to a national study carried out in 2012.^19^ Timely vaccination in our study was also very low, e.g. 20–22% of Roma children were immunized with DTP3 by the age of 12 months and 19–21% with MMR1 by the age of 24 months, while in the non-minority population of 6-year old children the respective coverage was 96% and 93%.^19^ Low MMR uptake is of particular public health concern in view of recent measles outbreaks in several European countries and among unvaccinated Roma children in Greece in 2006 and 2010.^27–29^

None of the children in our sample had been given a BCG vaccine before the age of 12 months, despite its longstanding administration at birth in high risk groups in countries with low burden of tuberculosis.^30^ Of note, the Roma population, both internationally and in Greece, have been shown to experience high incidence of tuberculosis, as well as of other vaccine preventable diseases, such as hepatitis B and hepatitis A.^14^^,^^31–33^

Very low vaccination coverage of Roma children is a consistent finding in studies conducted both internationally and in Greece. A well-designed subnational study in Bosnia-Herzegovina found lower coverage with some vaccines (e.g. 13% had been immunized with DTP3 by the age of 12 months) and higher with others (e.g. 85% vaccinated with BCG by the age of 12 months) compared with our study.^12^ Another well-designed study, carried out locally in Belgrade, Serbia, also showed very low vaccination coverage (e.g. 7% had age-appropriate full vaccination, 11% were vaccinated with MMR1 and DTP3, and 1.5% with BCG), but the age of the children studied (6–59 months) does not allow direct comparisons with our study.^9^ A recent systematic review of local vaccination studies among Roma children in Greece has invariably shown low vaccination coverage with all vaccines.^15^

As expected, we found that better living conditions were associated with higher immunization coverage and reported barriers to vaccination with lower coverage. Female gender was associated with lower coverage; this is possibly related to differences between the perceived role of sexes and/or considering school attendance of girls a lower priority compared with boys—as vaccination of Roma children in Greece is often related to attending school.^8^^,^^25^

Interestingly, more children from settlements where vaccination is usually provided by Medical-Social Centres started vaccination, presumably because of the appropriateness of a service close to the place of residence, but fewer continued to have ‘extended vaccination’. This is probably due to inadequate vaccine supplies and understaffing experienced in recent years as a consequence of the economic crisis.

## Conclusions and recommendations

Our study showed inadequate vaccination coverage of Roma children, much lower than that of the non-minority child population in Greece and the WHO targets.^34^ This is a serious public health challenge and should be systematically addressed as part of comprehensive social and development inclusion policies, which have increasingly been advocated in the past decade.^35^ Furthermore, in the midst of a deep and continuing economic recession in Greece, this group of children should be specifically aimed at with relevant social policies—including measures to promote vaccination—lest the inequality gap may widen.^36^

We found that primary care services close to Roma settlements contribute to vaccination uptake. It is vital to instigate vaccination programmes integrated into local primary care services, explicitly but not exclusively tailored to the needs of Roma communities. Continuous availability of vaccines and adequate staffing are crucial.

The lack of a sound evidence base to support Roma integration policies has been identified as a major obstacle towards this aim.^25^ In order to assess vaccination coverage of Roma children at a national level, we applied a study design involving stratified sampling and we identified the relative size of strata—used as weights in the analysis—through a consensus process. This approach proved to be feasible and may provide, both in Greece and in other countries of the European region, a means to obtain valid national data on important characteristics of the Roma population, and contribute to planning and evaluating interventions and policies.

## Supplementary data

Supplementary data are available at *EURPUB* online.

## Supplementary Material

Supplementary Data
