# Supplementary material for: Low vaccination coverage of Greek Roma children amid economic crisis: national survey using stratified cluster sampling
Source: Eur J Public Health. 2016 Sep 30;27(2):318–24. doi: 10.1093/eurpub/ckw179 (PMC5439213; doi:10.1093/eurpub/ckw179)
Supplement: Supplementary Data [file ckw179_Supp.zip › ckw179-suppl_data/ejph-2016-04-om-0316-File006.docx]

**Supplementary Table 1.** Summary of the Greek National Vaccination Programme (2011) for children 0–6 years old at the time of the present study.

| *Vaccine* | *Year in NVP** | *Total doses*  *0–6 years* | *1^st^ semester*  *of life* | *2^nd^ semester*  *of life* | *2^nd^ year*  *of life* | *Age 2–3 years* | *Age 4–6 years* |
| --- | --- | --- | --- | --- | --- | --- | --- |
| DTP | 1950s | 5 | 3 |  | 1 |  | 1 |
| IPV | 1960s | 4 | 2 | 1 | |  | 1 |
| BCG | 1960s | 1 | [1]† |  |  |  | 1 |
| MMR | 1989 | 2 |  |  | 1 |  | 1 |
| HepB | 1998 | 3 | 2 | 1 | |  |  |
| Hib | 2002 | 1 to 4‡ | 3 |  | 1 |  |  |
| MCVC | 2006 | 1 to 3‡ | 2 |  | 1 |  |  |
| PCV | 2006 | 1 to 4‡ | 3 |  | 1 |  |  |
| Var | 2006 | 2 |  |  | 1 |  | 1 |
| HepA | 2008 | 2 |  |  | 2 |  |  |
| * Year when vaccination for the respective disease(s) was introduced in the National Vaccination Programme (NVP), regardless of vaccine form or vaccination schedule. † It is recommended that BCG is given in the first days of life in children from high risk groups (including history of tuberculosis in the family, immigrants from endemic areas, Roma population); in the general population, BCG is recommended for the age of 6 years.  ‡ The recommended number of doses depends on the age at vaccination. If vaccination starts after the age of one (Hib, MCVC) or two years (PCV), one dose is recommended. For all three vaccines the NVP recommends early vaccination onset (2 months of age) and the maximum number of doses in the table.  Source: Ministry of Health and Social Solidarity, General Directorate of Public Health.^(17)^ | | | | | | | |
