# Supplementary material for: Low vaccination coverage of Greek Roma children amid economic crisis: national survey using stratified cluster sampling
Source: Eur J Public Health. 2016 Sep 30;27(2):318–24. doi: 10.1093/eurpub/ckw179 (PMC5439213; doi:10.1093/eurpub/ckw179)
Supplement: Supplementary Data [file ckw179_Supp.zip › ckw179-suppl_data/ejph-2016-04-om-0316-File007.docx]

**Supplementary Table 2.** Number of Roma settlements, number of children in the sample, and weights assigned in the analysis by stratum.

| *NUTS-1 region** | *Number of Roma settlements in the sample* | | | | *Number of children in the sample* | | | | *Weight assigned in the analysis* | | | |
| --- | --- | --- | --- | --- | --- | --- | --- | --- | --- | --- | --- | --- |
| *Settlement type*† | *ST1* | *ST2* | *ST3* | *Total* | *ST1* | *ST2* | *ST3* | *Total* | *ST1* | *ST2* | *ST3* | *Total* |
| Northern Greece | 3 | 3 | 3 | 9 | 23 | 28 | 28 | 79 | 0.219 | 0.162 | 0.113 | 0.494 |
| Central Greece | 4 | 4 | 4 | 12 | 28 | 34 | 27 | 89 | 0.078 | 0.092 | 0.134 | 0.304 |
| Attica | 2 | 2 | 2 | 6 | 18 | 19 | 27 | 64 | 0.043 | 0.045 | 0.090 | 0.178 |
| Crete/Aegean islands | 1 | 1 | 1 | 3 | 6 | 6 | 7 | 19 | 0.002 | 0.008 | 0.015 | 0.025 |
| Total | 10 | 10 | 10 | 30 | 75 | 87 | 89 | 251 | 0.342 | 0.307 | 0.352 | 1.00 |
| * NUTS-1: First level of Nomenclature of Units for Territorial Statistics.^(23)^  † Settlement types: Mainly houses (ST1); Mixture of houses and shacks/tents (ST2); Mainly shacks/tents (ST3) [modified from references (20) and (24)]. | | | | | | | | | | | | |
